# Evaluation of the Effects of High Uric Acid on Glucolipid Metabolism, Renal Injury and the Gut Microbiota in Diabetic Male Hamsters with Dyslipidemia

**DOI:** 10.3390/toxics13090751

**Published:** 2025-09-04

**Authors:** Liang He, Miao Miao, Qingxiangzi Li, Jufen Cheng, Rui Li

**Affiliations:** 1 Laboratory Animal Center, Zhejiang Academy of Agricultural Sciences, Hangzhou 310021, China; xueqiguorong@163.com (L.H.);; 2 Institute of Animal Husbandry and Veterinary Science, Zhejiang Academy of Agricultural Sciences, Hangzhou 310021, China; 3 Institute of Rural Development, Zhejiang Academy of Agricultural Sciences, Hangzhou 310021, China; 4 Institute of Agro-product Safety and Nutrition, Zhejiang Academy of Agricultural Sciences, Hangzhou 310021, China

**Keywords:** hyperuricemia, metabolic disorders, renal toxicity, intestinal flora, dysbiosis

## Abstract

The prevalence of hyperuricemia with elevated serum uric acid is increasing worldwide. However, the effects of high uric acid on diabetic patients with dyslipidemia and the mechanisms underlying these effects remain unexplored. This study aimed to develop a novel diabetic model of hyperuricemia and dyslipidemia in male hamsters to evaluate the effects of high uric acid on glucolipid metabolism, renal injury and the gut microbiota. Twelve healthy hamsters were randomly divided into two groups and fed with a normal diet and high-fat/cholesterol diet (HFCD), respectively. Twenty-four diabetic hamsters were randomly divided into four groups receiving a normal diet; HFCD; potassium oxonate (PO) treatment (intragastric PO at doses of 350 mg/kg and adenine at doses of 150 mg/kg with 5% fructose water); and PO treatment with HFCD, respectively. After 4 weeks, all animals were dissected for determining serum biochemical indicators, tissue antioxidant parameters, renal pathological changes, target gene expressions, fecal short-chain fatty acids content, and the gut microbiota composition. The results showed that a hamster model with hyperuricemia and dyslipidemia was successively established by the combination of PO treatment and HFCD, in which serum uric acid, glucose, triglyceride and total cholesterol levels reached 499.5 ± 61.96 μmol/L, 16.88 ± 2.81 mmol/L, 119.88 ± 27.14 mmol/L and 72.92 ± 16.62 mmol/L, respectively. PO treatment and HFCD had synergistic effects on increasing uric acid, urea nitrogen, creatinine levels, liver xanthine oxidase activity, plasminogen activator inhibitor-1 and transforming growth factor-β expressions, and the relative abundance of *Lleibacterium* (*p* < 0.05); in addition, they caused glomerular mesangial cells and matrix proliferation, protein casts and urate deposition. High uric acid was closely related to decreased antioxidant capacity; decreased renal vascular endothelial growth factor expression; increased acetic acid content; decreased butyric, propanoic, and isobutyric acid levels; decreased *Firmicutes* to *Bacteroidetes* ratios (*p* < 0.05); and altered epithelial integrity and structure of the gut microbiota in diabetic hamsters. The findings indicate that high uric acid affects the glucolipid metabolism, accelerates renal damage, and disrupts the balance of intestinal flora in diabetic animals, which provides a scientific basis for metabolic syndrome prevention and control in diabetes.

## 1. Introduction

As the end-product of purine catabolism, uric acid, which is most excreted by the kidney, is degraded by urate oxidase and hepatic enzymes to allantoin in most mammals [1]. Due to the evolutionary disruption of the gene encoding urate oxidase in humans, overproduction or underexcretion of uric acid leads to hyperuricemia [2]. The prevalence of hyperuricemia is greater than 20% in USA and 17.7% in China [3,4]. Many epidemiologic studies strongly suggest that high serum uric acid is closely related to an increased risk of chronic kidney disease, diabetes, and abnormal lipid metabolism [5,6,7]. Moreover, accumulating scientific evidence shows a close association of the onset and progression of hyperuricemia and alterations in the prevalence, distribution, and structural transformation of the intestinal microbiota [8]. Many clinical investigations have indicated that diabetes is often accompanied by dyslipidemia and hyperuricemia [9,10]. However, the effects of high uric acid on patients with diabetes and dyslipidemia and the underlying mechanisms have not been fully investigated.

Some researchers have shown that elevated uric acid levels are related to diabetes. Findings from Bhole and De Cosmo et al. indicated that high uric acid levels and hyperuricemia were significantly related to an increased risk of developing type 2 diabetes and insulin resistance, respectively [10,11]. Compared with no hyperuricemia, five-year prospective cohort study from China revealed that incident hyperuricemia and remittent hyperuricemia were associated with 48% and 35% greater risks, respectively, for the development of diabetes [12]. Jie et al. demonstrated that uric acid promoted pancreatic β-cell death, but alone was insufficient to induce diabetes [13]. Moreover, many epidemiological investigations have indicated that serum uric acid levels are positively correlated with lipid profiles in adult populations, and that hyperuricemia may be a risk factor for lipid metabolic disorders [14,15]. There is currently limited knowledge regarding the mechanism underlying diabetes with hyperuricemia and dyslipidemia at present. An important barrier is the lack of suitable animal diabetic models that can faithfully replicate the pathologic features of patients with diabetes, hyperuricemia, and hyperlipidemia.

Recently, uric acid has gained prominence as a key factor in multifactorial renal diseases, including acute renal injury and chronic renal disease [16]. Hyperuricemia has deleterious effects, including gout, nephrolithiasis, and renal damage [17]. Increasing evidences have indicated that lipid abnormalities contribute to glomerulosclerosis and progressive renal disease [18]. Diabetes also contributes to glomerular damage and renal arteriosclerosis in the progression of kidney damage [19]. Therefore, glycolipid metabolic disorders can result in cell apoptosis, lipid accumulation, mitochondrial damage, oxidative stress, and renal tubular epithelial cell injury. To our knowledge, there is limited information regarding the effects of high uric acid on kidney injury in patients with diabetes and dyslipidemia.

The gut microbiota, a complex microbial community colonizing the intestines, regulates many metabolic processes in the host including energy homeostasis, glucose metabolism and lipid metabolism [20]. Moreover, the gut microbiota is crucial for regulating uric acid metabolism, and dysregulation of the gut microbiota can disrupt uric acid homeostasis, leading to hyperuricemia [8]. The intricate relationship between uric acid metabolism and the gut microbiota involves a bidirectional interaction that can influence both the host’s gut environment and uric acid levels. Liu et al. identified 46 species of uric acid-degrading bacteria spanning four phyla: *Actinobacteria*, *Proteobacteria*, *Fusocbacteria* and *Firmicutes*, within the repository of human intestinal bacteria [21]. The composition of the gut microbiota is related to lipid metabolism, which is regulated mainly by nutrients, including fatty acids and sugars [22]. Intestinal flora imbalance also affects bile acid metabolism, intestinal permeability, the inflammatory response, and insulin resistance (IR), which are strongly correlated with diabetes [23]. Therefore, the role of high uric acid levels on the intestinal flora in patients with diabetes and the confounding factors of abnormal glucose and lipid metabolism must be determined.

Appropriate animal models are the foundation for further investigations of disease pathogenesis and new drug development. Several animal models, including rats and mice, have been established to explore the regulatory mechanisms underlying human metabolic diseases, such as hyperuricemia, dyslipidemia and diabetes [24,25]. Potassium oxonate (PO), a selectively competitive inhibitor of uricase, can substantially increase the concentration of uric acid. Hyperuricemia can be induced using PO which inhibits uricase activity in rodents [25]. The findings of Kobayashi et al. revealed that db/db mice presented significant obesity and fasting hyperglycemia when fed a high-fat diet (21% fat and 0.15% cholesterol) within 42 days of age, which was a good model for diabetic dyslipidemia [26]. As an antibiotic that causes pancreatic islet β-cell destruction, low-dose streptozotocin (STZ) is widely used to induce diabetes in mice and rats [27]. Compared with mice and rats, hamsters are better at reproducing hyperlipidemia, which is very similar to the hepatic lipid metabolism and cholesteryl ester transfer protein activities of humans [28]. The effects of dietary cholesterol on blood lipid profiles are similar between hamsters and humans.

Many clinical investigations have shown that the prevalence of hyperuricemia in males is significantly higher than that in females [29,30]. Therefore, this study aimed to establish a novel diet-induced diabetic model with hyperuricemia and dyslipidemia in male Golden Syrian hamsters to investigate the biochemical and histological changes, and evaluate the effects of high uric acid on glucolipid metabolism, renal injury and the gut microbiota in hamsters. Furthermore, the synergistic effects of hyperuricemia, hyperlipidemia, and diabetes were explored in hamsters. These findings may provide novel insights into the interaction effects of multiple risk factors on metabolic syndrome in humans.

## 2. Materials and Methods

### 2.1. Animal Experimental Design

Forty-two healthy male Golden Syrian hamsters (10 weeks old, 163 ± 7.43 g) were obtained from Vital River Laboratory Animal Technology Company (Beijing, China). All animals were raised in a standard environment (temperature: 20–24 °C, relative humidity: 45–55% and light–dark cycles: 12 h) with freely accessible food and water. The animal experiments were approved by the Institutional Animal Care and Use Committee (IACUC)-Zhejiang Academy of Agricultural Sciences (2022ZAASLA58). STZ (>98% purity, CAS: 18883-66-4) was purchased from Sigma-Aldrich Chemical Company (St. Louis, MO, USA) and dissolved in 0.05 M citrate buffer (pH 4.5). PO (>98% purity, CAS: 2207-75-2) and adenine (>99% purity, CAS: 73-24-5) were purchased from Shanghai Macklin Biochemical Technology Co., Ltd (Shanghai, China). and resuspended in a 0.5% sodium carboxymethyl cellulose (CMC-Na) solution. Commercial standard rodent chow, and high-fat/cholesterol (15% fat, 0.5% cholesterol) diet (HFCD) were provided by Keao Xieli (Tianjin) Feed Co., Ltd. (Tianjin, China).

**Figure 1:**
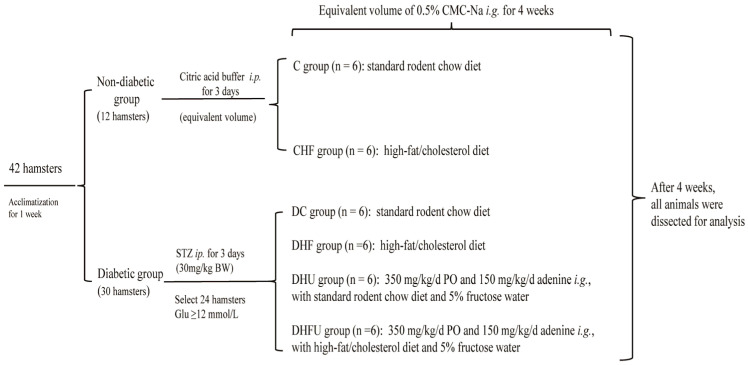
Experimental animal design.

Diabetes was induced in thirty hamsters by the intraperitoneally injection of STZ (30 mg/kg) once daily for 3 consecutive days. After ten days, 24 hamsters with a fasting blood glucose (Glu) concentration (>12 mmol/L) were randomly divided into 4 groups (*n* = 6): DC group, standard diet; DHF group, HFCD; DHU group, PO treatment (intragastric PO at doses of 350 mg/kg and adenine at doses of 150 mg/kg with 5% fructose water) with a standard diet; and DHFU group, PO treatment with HFCD. Twelve healthy hamsters were randomly divided into two groups (*n* = 6): CHF group, HFCD; and C group, standard diet. Hamsters in the C, DC, DHF and CHF groups were intragastrically administered the same volume of CMC-Na water (0.5%). The experimental animal design is presented in Figure 1.

### 2.2. Measurements of Body Weight (BW), Organ Index, and Serum Biochemical Indicators

BW were measured daily after ten days of STZ injection. The hamsters were anesthetized using isoflurane before they were sacrificed and weighed. Tissues (kidney and liver) were sampled and weighed for organ index calculation (the ratio of the kidney and liver weights to the BW), which was an important indicator of visceral health. Blood samples from the venous retroorbital plexus of hamsters were collected, centrifuged for 15 min at 3000× *g* and stored at −20 °C. Glu were measured throughout the experiment (before STZ injection; at ten days after STZ injection; at the experimental endpoint). Serum uric acid, Glu, total cholesterol (TC), triglyceride (TG), creatinine (CRE), urea nitrogen (BUN), alkaline phosphatase (ALP), aspartate transaminase (AST) and alanine transaminase (ALT) levels were detected using an automatic biochemistry analyzer (Leidu Life Technology, Shenzhen, China). Insulin was assayed with a hamster insulin ELISA kit (BlueGene Biotech Co., Ltd., Shanghai, China).

### 2.3. Measurement of Liver Xanthine Oxidase (XOD) and Kidney Antioxidant Parameters

At the experimental endpoint, liver and kidney tissues were dissected quickly and parts of the two tissues were frozen in liquid nitrogen and stored at −80 °C for further XOD and antioxidant parameter analysis. XOD, total superoxide dismutase (T-SOD), glutathione peroxidase (GPX-PX) and malondialdehyde (MDA) were detected using commercial assay kits (Nanjing Jian Cheng Bioengineering Institute, Nanjing, China).

### 2.4. Renal Histological Analysis

The right kidney was placed in 4% paraformaldehyde solution for 48 h. After trimming and tissue processing, kidney tissue was embedded in paraffin, and cut into 5 μm thick sections for hematoxylin and eosin (H&E) and Periodic Acid-Schiff (PAS) staining. Finally, histopathological observations were performed using a microscope (Leica DM2500, Wetzlar, Germany).

### 2.5. Total RNA Extraction and Quantitative Polymerase Chain Reaction (qPCR)

A portion of the kidney tissue was removed from the killed hamsters and quickly placed in liquid nitrogen for total RNA extraction using RNA extraction reagents (Servicebio, Wuhan, China). cDNA was reverse transcribed from 2 μg of total RNA with a first strand cDNA synthesis kit (Servicebio, Wuhan, China). The transcript levels of the target genes (TNF-α, SREBP-1c, PAI-1, IL-6, TGF-β, and VEGF) were evaluated by qPCR with 2 × SYBR Green qPCR Master Mix (Servicebio, Wuhan, China). β-actin was used as a reference to normalize the mRNA levels of the chosen genes. The relative expression of the target genes was calculated using the 2^−ΔΔCt^ method. All primer sequences are listed in Table 1.

### 2.6. Fecal Short-Chain Fatty Acid (SCFA) Measurement

SCFA levels were detected using methods from Li et al. with minor modifications [31]. Briefly, 100 mg of thawed fecal sample was added to 1.0 mL of water and 0.2 mL of crotonic acid–metaphosphoric acid mixture (64.6 mg of crotonic acid and 2.5 g of metaphosphoric acid dissolved in 10.0 mL of water) and then acidified for 24 h at −40 °C. The mixture was centrifuged at 4 °C for 3 min at 13,000× *g*, filtered through a 0.22 μm PVDF filter, and injected into a gas chromatograph (Shimadzu, Japan) for determination of the SCFAs under the following conditions: inlet and FID detector temperatures of 250 °C and a carrier gas flow rate of 20 mL/min with a 10:1 split ratio. In addition, the column temperature began at 80 °C, gradually increased to 190 °C (10 °C/min) with a hold for 0.5 min, and then increased to 240 °C (40 °C/min) with a hold for 4 min. Crotonic acid was used as the internal standard.

### 2.7. Gut Microbiota Analysis

At the experimental endpoint, fresh fecal samples from all the hamsters were collected and immediately stored at −80 °C for 16S rRNA gene sequencing. The DNA of the fecal samples was extracted using a fecal genome DNA extraction kit (Tiangen Biotech Co., Ltd., Beijing, China). The V3-V4 variable region of the 16S rRNA gene was subsequently amplified using bacterial primers (341F 5′-CCTAYGGGRBGCASCAG-3′ and 806R 5′-GGACTACNNGGGGTATCTAAT-3′). The purified PCR products were used for library construction and sequenced on the Illumina NovaSeq PE250 platform (San Diego, CA, USA). The raw sequences were quality filtered with Fastp software (v 0.19.6) and merged using Flash software (v 1.2.11). The qualified sequences were subsequently denoised using the DADA2 plugin in QIIME2 (v 2020.2) to obtain amplicon sequence variants (ASVs).

### 2.8. Statistical Analysis

All results are presented as the means ± standard deviations (SDs). GraphPad Prism 8.0 (Graphpad Software Inc., San Diego, CA, USA) and SPSS 22.0 (IBM, Armonk, NY, USA) were used for graphical presentation and statistical analysis. Levene’s test was used to determine whether the data were homogeneous in variance. Data with equal variances were analyzed by one-way ANOVA coupled with Tukey’s test. Data with unequal variances were analyzed by Kruskal–Wallis Test. The *p* value cutoff was set as 0.01 or 0.05.

## 3. Results

### 3.1. Induction of Diabetes, Hyperuricemia and Dyslipidemia in Hamsters

After PO treatment for 4 weeks, the serum uric acid levels in the DHU and DHFU groups were significantly higher than those in the DC and DHF groups, respectively, indicating that the PO treatment developed severe hyperuricemia in diabetic hamsters (Table 2). Moreover, the serum uric acid level detected in the DHFU group was significantly greater than that in the DHU group (*p* < 0.05). In hamsters fed HFCD (CHF, DHF and DHFU), the serum TG and TC levels significantly increased in comparison with those in the C and DC groups, indicating that hamsters in the DHF and DHFU groups developed severe dyslipidemia (Table 2). However, there were no marked differences in TG or TC levels between the DHF and DHFU groups. The blood samples from the DHFU group had a milky appearance with significantly elevated serum uric acid, Glu, TG and TC levels, and decreased insulin levels, indicating the successful establishment of a diabetic hamster model with hyperuricemia and dyslipidemia (Table 2). These results indicated that the combination of PO treatment and HFCD had synergistic effects on increasing serum uric acid levels in diabetic hamsters developing hyperuricemia. In addition, high uric acid and dietary fat/cholesterol had non-synergistic effects on increasing serum Glu levels in diabetic hamsters.

### 3.2. Effects of High Uric Acid on BW and Organ Index in Diabetic Hamsters

As shown in Table 3, the BWs of the DHU and DHFU groups were significantly lower than those of the C group and CHF group (*p* < 0.01). However, no significant differences in BW were found among the DHF, DHU and DHFU groups, which indicating that elevated uric acid alone had a negligible effect on weight loss in diabetic hamsters. The BW of hamsters in the CHF group was significantly greater than that in the DHF, DHU and DHFU groups (*p* < 0.01). Table 3 shows that the kidney indices of all diabetic groups were greater than those of the C and CHF groups (*p* < 0.01). However, no marked differences in the kidney indices were detected among the diabetic groups. The liver index of the DHFU group was significantly greater than those of the C, CHF, DC and DHU groups (*p* < 0.01). These result suggested that elevated uric acid alone had a negligible effect on the organ indices of diabetic hamsters.

### 3.3. Effects of High Uric Acid on CRE, BUN, ALT, AST and ALP in Diabetic Hamsters

As important clinical indices of renal function, elevated CRE levels likely indicate impaired kidney function and elevated BUN levels are detected in the decompensation period of renal insufficiency [32]. As enzyme biomarkers, serum ALT, AST, and ALP levels are used to evaluate the structural integrity of and damage to the liver. An increase in these indicators suggests that hepatocytes are damaged to a certain extent. Table 4 showed that the CRE levels in the DHFU and DHF groups were significantly greater than those in the C, DC and DHU groups (*p* < 0.01). The CRE level in the DHFU group was significantly greater than that in the DHF group (*p* < 0.01). The BUN level in the DHFU group was significantly greater than that in the C and DHU groups. The ALT level in the DHFU group was significantly greater than that in the C and DC groups, and no significant differences in ALT were found between the CHF and DHFU groups (Table 4). The AST and ALP levels in the DHFU group were obviously greater than those in other five groups (*p* < 0.01). These results suggested that high uric acid and HFCD synergistically effects increased serum AST, ALT, and ALP levels in the DHFU group.

### 3.4. Effects of High Uric Acid on Liver and Kidney Enzyme Levels to Analyze Oxidative Stress in Diabetic Hamsters

Hepatic XOD is a flavoprotein that catalyzes the oxidation of hypoxanthine and xanthine to uric acid, and inhibition of XOD activity can decrease the production of uric acid [33]. T-SOD, GSH-PX and MDA play important roles in oxidative stress in animals. Table 5 showed that PO treatment increased the XOD activity of the liver in the DHFU group compared with that in the C, CHF, DC, and DHU groups (*p* < 0.01). A HFCD (administered to the CHF, DHF, and DHFU groups) induction increased liver XOD activity when compared to that in the C group (*p* < 0.01). These results indicated that the high uric acid and HFCD synergistically increased the activity of XOD in the setting of diabetes. As shown in Table 5, when uric acid was increased by PO induction, hamsters in the DHFU group developed oxidative stress with increased MDA and decreased T-SOD and GSH-PX.

### 3.5. Effects of High Uric Acid on Renal Histology in Diabetic Hamsters

**Figure 2:**
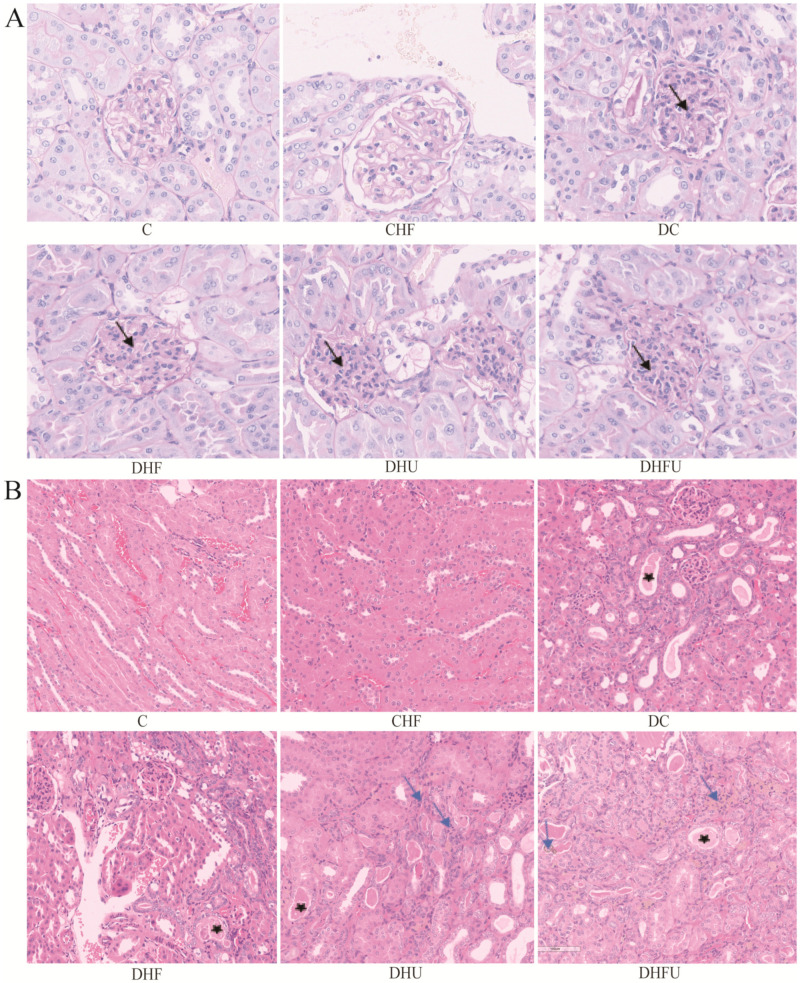
Microscopic observation of the kidney using PAS staining ((**A**), 400×) and H&E staining ((**B**), 200×). Note: black arrows indicate glomerular mesangial cells and mesangial matrix proliferation, black stars indicate renal tubular dilatation and casts, and blue arrows indicate urate deposition and inflammatory cell infiltration.

Histological observation of the kidney tissues from each group of hamsters via PAS and H&E staining were shown in Figure 2A,B. There were no obvious glomerular changes in nondiabetic animals (C and CHF groups), and diabetic animals presented with glomerular mesangial cells and mesangial matrix proliferation, especially in the DHFU group (indicated by the black arrows in Figure 2A). Figure 2B showed that the epithelial cells of the renal tubules were intact and clear and that the renal interstitial structure was normal in the nondiabetic groups (C and CHF). However, the renal tubules in diabetic animals presented varying degrees of damage. The renal damage of the animals in the DC and DHF groups was minimal, with only renal tubule dilation, degeneration, and protein casts observed (indicated by the black star). Slight tubule dilation and protein casts were observed in the renal tissues (indicated by the black star) in the DHU group, and urate deposition and inflammatory cell infiltration were also found (indicated by the blue arrows). Notably, the renal tissue in the DHFU group was the most severely damaged, with moderate tubular degeneration, tubular atrophy, inflammatory cell infiltration, urate deposition, and protein casts (indicated by the blue arrows).

### 3.6. Effects of High Uric Acid on Renal Gene Expression in Diabetic Hamsters

**Figure 3:**
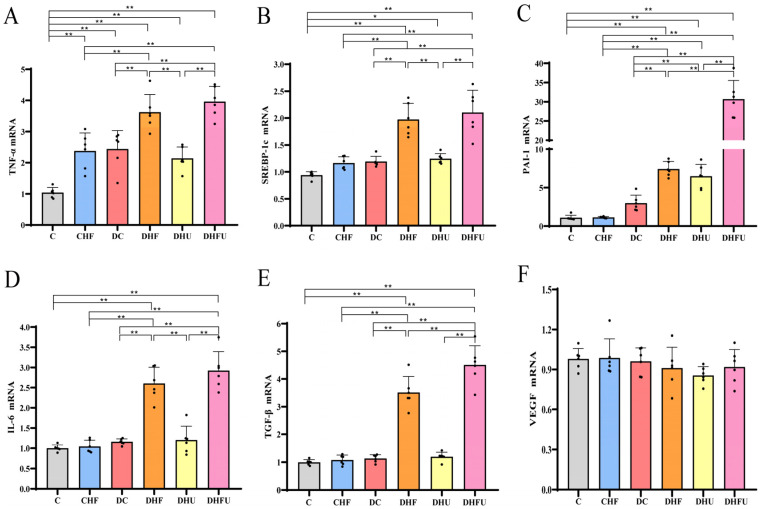
Effects of high uric acid on (**A**) TNF-α, (**B**) SREBP-1c, (**C**) PAI-1, (**D**) IL-6, (**E**) TGF-β, and (**F**) VEGF expression (*n* = 6). Note: * *p* < 0.05, ** *p* < 0.01, and black dot (·) represents a data point.

In the DHFU group, TNF-α mRNA expression was significantly upregulated compared with that in the nondiabetic groups (C and CHF groups), DC, and DHU groups (Figure 3A). However, no marked differences in TNF-α mRNA expression were detected between the DHFU and DHF groups (*p* > 0.05). SREBP-1c mRNA expression in the DHFU and DHF groups was significantly greater than that in the DC group, whereas SREBP-1c mRNA expression was not significantly different between the DHFU and DHF groups (Figure 3B). Figure 3C showed that PAI-1 mRNA expression in the DHFU group was also significantly upregulated compared with that in the other five groups (C, CHF, DC, DHF and DHU groups). Figure 3D showed that renal IL-6 mRNA expression in the DHFU group was markedly upregulated compared with that in the other four groups (C, CHF, DC, and DHU groups). Compared with that in the DHF and DHU groups, the renal mRNA expression of TGF-β in the DHFU group was significantly greater (Figure 3E). These results indicated that high uric acid and HFCD synergistically up-regulated renal gene expression of PAI-1 and TGF-β in diabetic hamsters. Figure 3F showed that there was no significant change in renal VEGF mRNA expression in any group (*p* > 0.05).

### 3.7. Evaluation of the High Uric Acid Effects on Fecal SCFAs in Diabetic Hamsters

**Figure 4:**
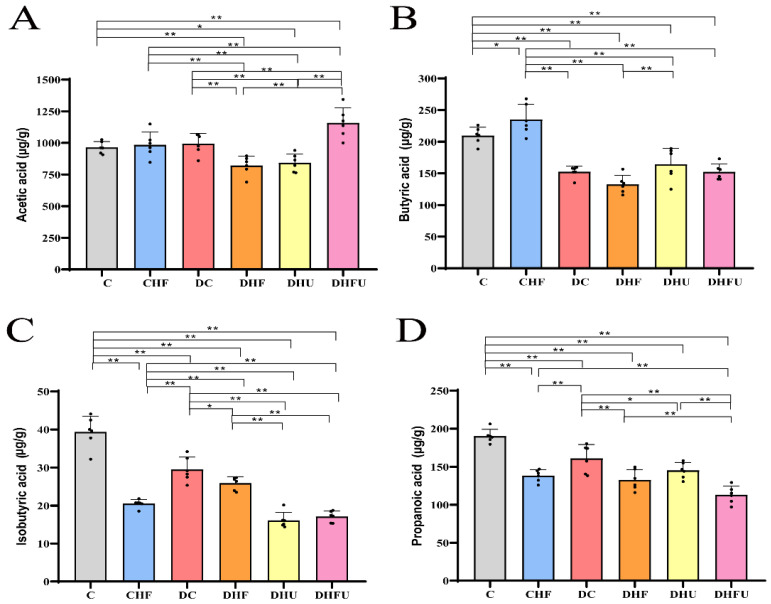
Effects of high uric acid concentrations on (**A**) Acetic acid, (**B**) Butyric acid, (**C**) Isobutyric acid, and (**D**) Propionic acid levels in diabetic hamsters. (*n* = 6). Note: * *p* < 0.05, ** *p* < 0.01, and black dot (·) represents a data point.

Fecal SCFAs, including acetic acid, butyric acid, isobutyric acid, and propionic acid, were detected using GC–MS. As shown in Figure 4A, the fecal acetic acid level in the DHFU group was significantly greater than that in the other groups (C, CHF, DC, DHF, and DHU groups) (*p* < 0.01). The butyric acid levels in the four diabetic groups (DC, DHF, DHU, and DHFU groups) were significantly lower than that in the CHF and C groups; however, the butyric acid levels did not differ among the DC, DHU, and DHFU groups (Figure 4B). Figure 4C showed that the isobutyric acid levels in the two high uric acid groups (DHU and DHFU groups) were significantly lower than that in the other four groups (C, CHF, DC, and DHF groups), and no significant differences in isobutyric acid levels were detected between the DHU and DHFU groups. As shown in Figure 4D, the propanoic acid level in the DHFU group was significantly lower than that in the other diabetic groups (*p* < 0.01).

### 3.8. Evaluation of the High Uric Acid Effects on the Gut Microbiota in Diabetic Hamsters

**Figure 5:**
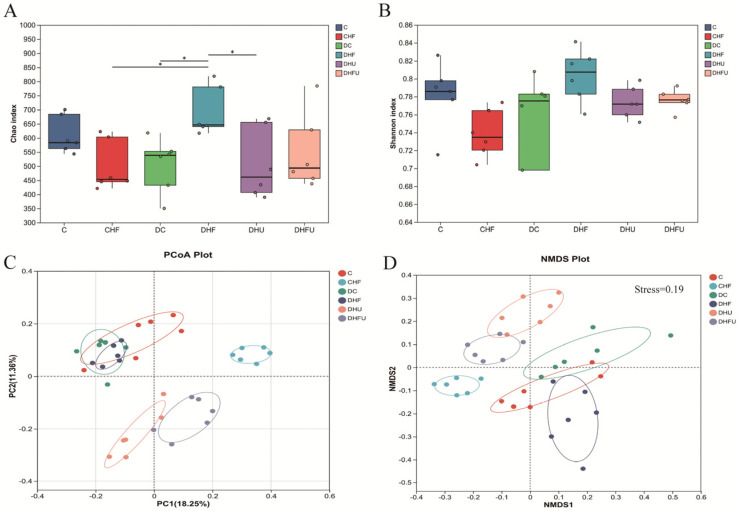
Effects of high uric acid concentrations on gut microbiota diversity in hamsters. (**A**) Chao index, (**B**) Shannon index, (**C**) PCoA and (**D**) NMDS. * *p* < 0.05.

As shown in Figure 5A, the diversity analysis indicated that the Chao index in the DHF group was significantly greater that in the CHF, DC and DHU groups (*p* < 0.05). The Chao index reflects richness, indicating that a HFCD in diabetic hamsters could significantly increase the richness of the intestinal flora. However, the Chao index of the DHU group was not different from that of the corresponding control groups (C and DC groups), indicating that elevated uric acid induced by PO treatment hardly improved the richness of the intestinal flora. The Shannon index reflects the diversity of bacterial communities, and there were no significant differences among the groups (*p* > 0.05) (Figure 5B), indicating that both PO treatment and the HFCD did not affect the intestinal flora diversity in hamsters. When the differences in the distribution of microbial communities were compared through principal coordinate analysis (PCoA) and nonmetric multidimensional scaling (NMDS), the results revealed that the gut microbiota structure in the fecal samples changed in diabetic hamsters with hyperuricemia and dyslipidemia, as the DHFU group presented different clusters of samples than the other groups (C, CHF, DC and DHF groups) (Figure 5C,D). After PO administration, the clusters in the DHU and DHFU groups were obviously separated from those in the DC and DHF groups, indicating that high uric acid induced by PO treatment could also alter the structure of the gut microbiota.

**Figure 6:**
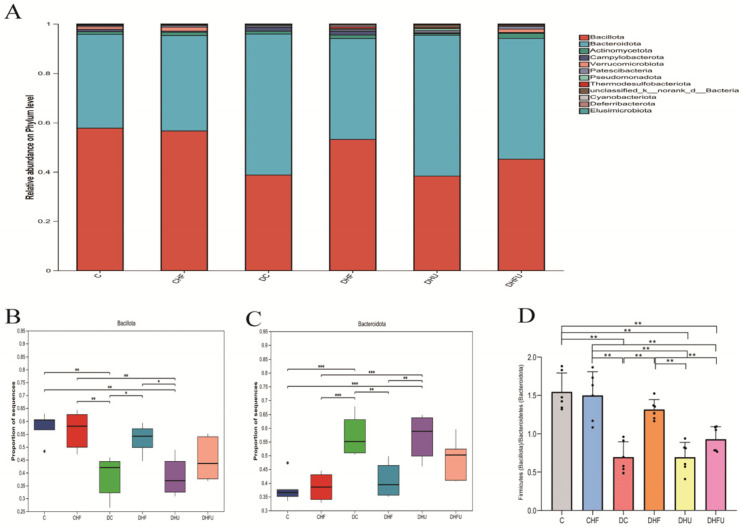
Effects of high uric acid on the components of the gut microbiota in hamsters at the phylum level. (**A**) Relative abundance of the gut microbiota; differences in (**B**) *Bacillota* and (**C**) *Bacteroidota* among groups; (**D**) ratio of *Firmicutes* (*Bacillota*) to *Bacteroidetes* (*Bacteroidota*). * *p* < 0.05, ** *p* < 0.01, and *** *p* < 0.001.

As shown in Figure 6A, a total of 10 microorganisms with the highest abundance at the phylum level, mainly *Firmicutes* (*Bacillota*) and *Bacteroidetes* (*Bacteroidota*), were present in the fecal samples of all the groups. A greater abundance of *Bacillota* and a lower abundance of *Bacteroidota* were detected in the CHF and DHF groups than in the DC group (*p* < 0.05 or 0.01), whereas PO treatment diminished this trend (Figure 6B, C), indicating that elevated uric acid had the opposite effect of the intake of high-fat/cholesterol in altering the gut microbiota at the phylum level in diabetic hamsters. An increasing ratio of *Firmicutes* to *Bacteroidetes* (F/B) was more likely to cause obesity [34]. Compared with those of healthy hamsters, the average F/B values of hyperuricemic hamsters (DHU and DHFU groups) were significantly lower (*p* < 0.01) (Figure 6D). However, there was no significant difference in the F/B value between normal diabetic hamsters (DC group) and hyperuricemic hamsters (DHU and DHFU groups), indicating that high uric acid induced by PO treatment has a negligible effect on obesity in diabetic hamsters.

**Figure 7:**
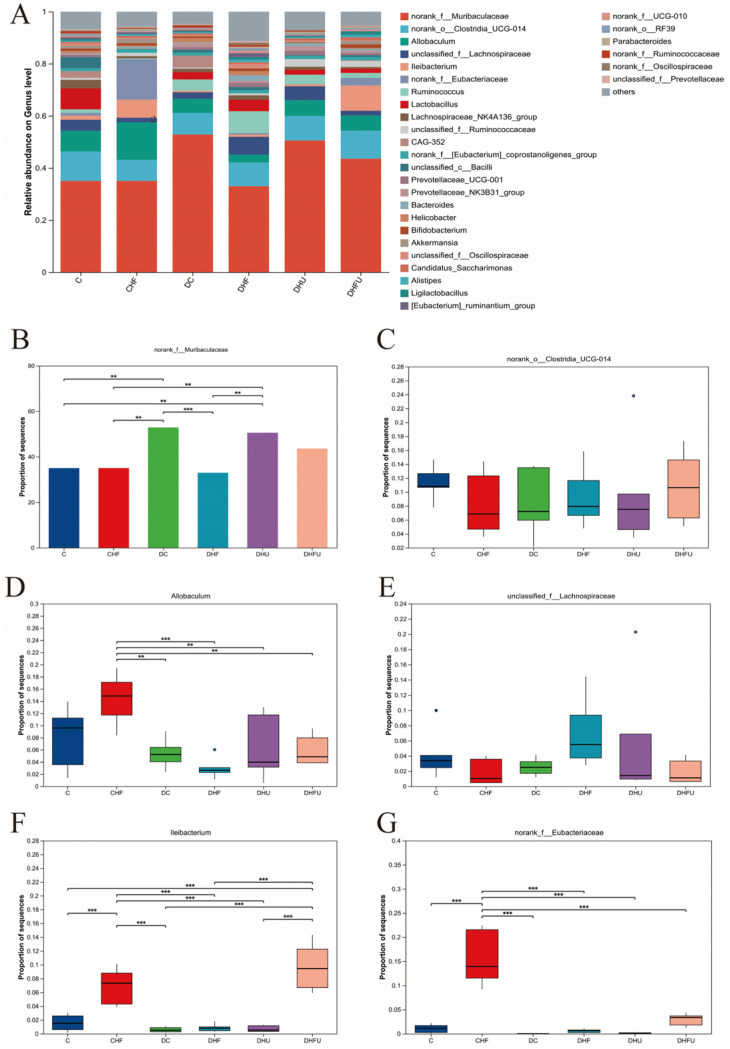
Effects of high uric acid on the components of the gut microbiota in hamsters at the genus level. (**A**) Relative abundance of the gut microbiota; (**B**–**G**) Relative abundance of the top six species. ** *p* < 0.01, and *** *p* < 0.001.

As shown in Figure 7A, the dominant bacteria among the top 20 microorganisms at the genus level in terms of abundance in hamster feces mainly included *norank_f_Muribaculaceae*, *norank_o_Clostridia_UCG-014, Allobaculum*, *unclassified_f_Lachnospiraceae*, *lleibacterium,* and *norank_f_Eubacteriaceae*. As shown in Figure 7B, the enrichment of *norank_f_Muribaculaceae* in diabetic hamsters was elevated in the high uric acid group (DHU group) by PO treatment, whereas the dietary high-fat/cholesterol clearly decreased the enrichment of *norank_f_Muribaculaceae*. The enrichment of *norank_o_Clostridia_UCG-014* did not significantly differ among all the groups (*p* > 0.05), indicating that elevated uric acid caused by PO treatment had no effect on its enrichment (Figure 7C). Figure 7D showed that the enrichment of *Allobaculum* in the CHF group was significantly greater than that in all the diabetic groups (*p* < 0.01). However, no significant difference in the enrichment of *unclassified _f_Lachnospiraceae* was found among all the groups, indicating that high uric acid elevated by PO had no effect on its enrichment (Figure 7E). Figure 7F showed that the enrichment of *lleibacterium* in the DHFU group was significantly greater than that in the DC, DHF, and DHU groups (*p* < 0.01). Figure 7G showed that the enrichment of *noran_f_Eubacteriaceae* in the CHF group significantly increased than all diabetic groups (*p* < 0.01).

**Figure 8:**
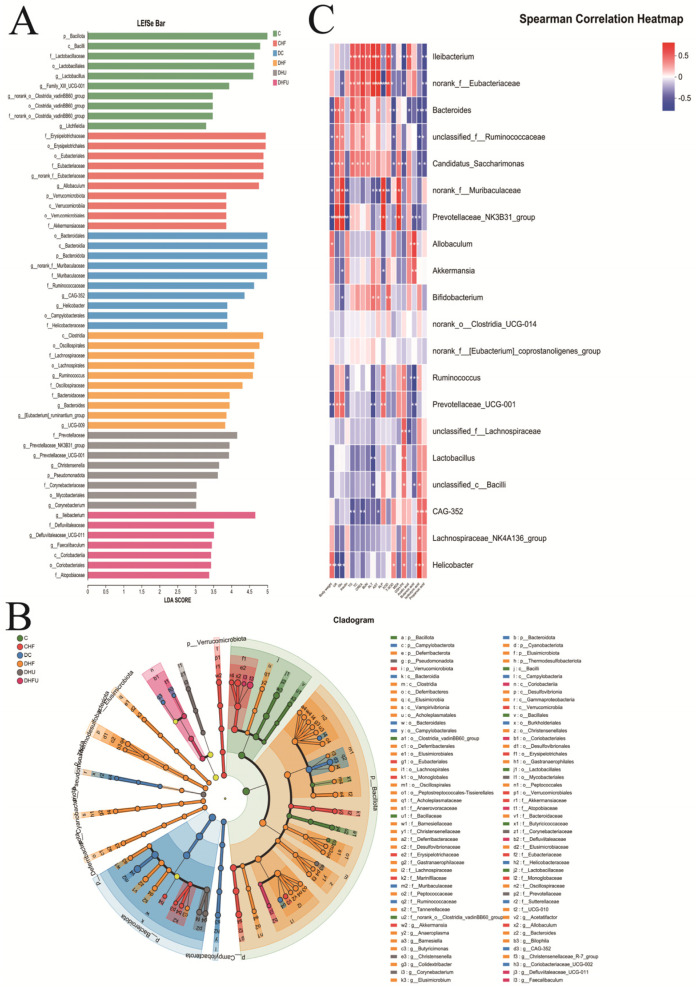
LEfSe analysis and correlation analysis. (**A**) LDA discriminant bar graph; (**B**) Evolutionary branching graph; (**C**) Correlations between the abundance of the gut microbiota and metabolic indicators in hamsters; red-white-blue color: positive correlation-no correlation-negative correlation. * *p* < 0.05, ** *p* < 0.01, and *** *p* < 0.001.

As shown in Figure 8A,B, LDA effect size (LEfSe) analysis (LDA score > 3) revealed that the characteristic microbiotas of the healthy hamsters (C group) were *p_Bacillota*, *c_Bacilli*, *f_Lactobacillaceae*, *f_norank_o_Clostridia_vadinBB60_group, o_Lactobacillates*, *o_Clostridia_vadinBB60_group*, *g_Lactobacillus*, *g_norank_o_Clostridia_vadinBB60_group*, *g_Family_XIII_UCG-001*, and *g_Litchfieldia* at the phylum and genus levels. The characteristic microbiota of the diabetic hamsters (DC group) included *o_Bacteroidales*, *p_Bacteroidota*, *c_Bacteroidia*, *g_norank_f_Muribaculaceae*, *f_Ruminococcaceae*, *f_Muribaculaceae*, *g_CAG-352*, *g_helicobacter*, *o_Campylobacterales*, and *f_Helicobacteraceae* at the phylum and genus levels. The characteristic microbiota of the DHFU group included *g_lleibacterium*, *f_Defluviitaleaceae*, *g_Defluviitaleaceae_UCG-001*, *g_Faecalibaculum*, *c_Coriobacteriia*, *o_Coriobacteriales* and *f_Atopobiaceae*. In addition, Spearman’s correlation analysis revealed that the abundance of *lleibacterium*, the dominant bacterium in the DHFU group, was positively correlated with the TG, TC, CRE, BUN, ALT, AST, XOD, and acetic acid levels and negatively correlated with the ALP, T-SOD, GSH-PX and propanoic acid levels (Figure 8C). Moreover, Spearman’s correlation analysis revealed that the uric acid levels in hamsters were positively associated with *Bacteroides*, *unclassified _f_Ruminococcaceae*, *Candidatus_Saccharimonas*, *norank_f_Muribaculaceae*, *Prevotellaceae_NK3B31_group*, and *Prevotellaceae_UCG-001* levels, and negatively associated with *Helicobacter* levels.

## 4. Discussion

Currently, most animal models of hyperuricemia are established in rodents, including rats and mice, which have advantages such as the ease of feeding, administration, and reproduction. Based on the evolutionary loss of uricase in humans and its secretion in rodents, the commonly used animal model of hyperuricemia should eliminate the difference in uricase between humans and model animals to better simulate the rise of human blood uric acid levels. Adenine is a form of nitrogen with a final metabolite of uric acid [24]. In some studies, PO, an inhibitor of uricase, was commonly used in combination with adenine to establish mouse models of hyperuricemia [35,36]. High-fructose also significantly increased serum uric acid levels and risk of hyperuricemia [37,38]. Thus, our modeling strategy was to combine the PO with adenine and high-fructose intake was chosen to inhibit uricase activity and increase the intake of purines in hamsters, thereby raising the uric acid levels. Moreover, low-dose STZ and short-term HFCD had been used for inducing a diabetic hamster model with dyslipidemia in our previous study [39]. Finally, combination of PO treatment, HFCD and low-dose STZ was used to establish a novel diet-induced diabetic model with hyperuricemia and dyslipidemia in hamsters in our study. To the best of the authors’ knowledge, this was the first report that a novel diet-induced diabetic animal model with hyperuricemia and dyslipidemia was successively established with the advantages of more effective cost, easier development and better simulation for the pathogenesis of diabetes companied with hyperuricemia and dyslipidemia. As an important complement to existing animal models, this novel diabetic hamster model replicated the physiological features of some specific population groups at the early stage of diabetes.

In comparison with a previous study from hyperuricemic mice induced by oral gavage of a mixture of PO (1000 mg/kg) and adenine (100 mg/kg) for 3 weeks, serum uric acid levels in hyperuricemic hamsters were higher than this reported value in mice [36]. Serum uric acid levels in hyperuricemic hamsters were over two times the previous value in mice drinking 15% high-fructose water for 8 weeks [38]. Meanwhile, diabetic model with hyperuricemia and dyslipidemia also developed severe hypertriglyceridemia and hypercholesterolemia with significantly increased plasma TC and TG, which was consistent with previous reports in mice feeding a diet containing high fat (40% fat) and high cholesterol (1.25% cholesterol) [40]. Our findings revealed that dietary high-fat/cholesterol elevated uric acid in diabetic hamsters developing hyperuricemia, which was in accordance with a previous report that elevated TG or TC levels were positively associated with hyperuricemia in 298,891 participants in a study from China in 2024 [41]. Increased dietary cholesterol absorption from the small intestine may lead to hypercholesterolemia in hamsters. Moreover, increased exogenous dietary triglyceride absorption or decreased TG uptake in peripheral tissues may attribute to the development of severe hypertriglyceridemia in hamsters. The underlying mechanism behind these findings may be attributed to dyslipidemia escalating the risk of hyperuricemia by triggering or intensifying processes involving purine metabolic disturbances. Obviously, reduction in high fat and high cholesterol intake may be a useful strategy to protect against hyperuricemia in diabetic patients. However, the intake of a high-fat/cholesterol diets can induce obesity, the metabolic relationship between obesity and hyperuricemia, including the onset of gout, is obscure [42]. The study from Wardhana et al. indicated that high uric acid levels lead to oxidative stress by increasing reactive oxygen species production, resulting in reduced insulin sensitivity and production from pancreatic islet cells [43]. However, our findings revealed that elevated uric acid levels were not positively related to high Glu and low insulin levels in STZ-induced diabetic hamsters, which indicated that uric acid accelerated but did not cause diabetes by inhibiting islet β-cell survival.

Findings from the most significantly increasing the kidney index in the DHFU group, showed that high uric acid and lipid levels jointly promoted severe damage to the kidney in diabetic animals, which was consistent with the results of significant renal hypertrophy in hyperuricemic rats reported by Cheng et al. [24]. However, a comparison of the liver indices of the DHF (most significantly high) and DHU (significantly low) groups revealed that dietary high-fat/cholesterol, not high uric acid, played a possible role in the hepatic injury induced by cholesterol accumulation during diabetes progression, which was consistent with hypotheses on effects of dietary fatty acids and cholesterol excess on liver injury in rats [44]. Li’s study of hyperuricemic rat models revealed that the uric acid level was not significantly correlated with the Cre or BUN levels [45]. Recent studies have also shown that increased serum UA directly promotes oxidative stress [46]. Sha et al. reported the significantly low expression levels of GSH-PX and T-SOD and high expression levels of MDA in the kidneys of a rat model of diabetic nephropathy [47]. Our findings of decreased GSH-PX and T-SOD and increased MDA in the DHFU group also revealed that the antioxidant capacity of the hamster body was weakened, indicated by oxidative damage, which might stimulate renal inflammation and damage.

Clinical investigation from Fan et al., revealed that high uric acid levels in patients with nephropathy are associated with glomerulosclerosis and tubulointerstitial injury [48]. Our findings revealed that high uric acid levels lead to renal injury in diabetic hamsters with tubular degeneration and atrophy, which was in agreement with the histopathological features of renal biopsies from hyperuricemic individuals. However, no obvious glomerulosclerosis was found in diabetic hamsters with hyperuricemia, which might be due to the experimental period and the age of the hamsters. In humans, severe renal injury often occurs at the late stage of the progression of hyperuricemia. The UOX-KO rats prepared by Wu et al. using the urate oxidase (UOX) gene knockout presented renal swelling and polycystic changes at 56 days of age, interstitial urate crystal deposition, dilation and fibrosis of renal tubules, fibrosis of glomeruli, and infiltration of macrophages at 112 days of age [49]. We found that the pathological features of diabetic hamsters with hyperuricemia and dyslipidemia included urate deposition and renal tubular dilation, degeneration and atrophy, which were similar to those observed in UOX-KO rats, although no fibrotic damage was observed.

Abrass et al. reported that lipids could stimulate mesangial cells to proliferate, produce excess basement membrane material and caused the development of sclerotic glomerular lesions [50]. In agreement with Abrass’s study, we also found that the TNF-α, SREBP-1c, PAI-1, IL-6, and TGF-β mRNA levels were significantly increased in diabetic hamsters with dyslipidemia. However, hyperuricemic diabetic hamsters also presented significantly increased PAI-1 mRNA levels, which contributed to diabetic nephropathy by increasing renal extracellular matrix production and accelerated renal injury in diabetic hamsters. No significant differences in the IL-6, SREBP-1c, or TNF-α mRNA levels were detected between the DHFU and DHF groups, indicating that the renal mRNA expression of these genes was affected mainly by lipid accumulation, not high uric acid. Increased expression of VEGF in the kidney was found in a db/db mice model of type 2 diabetes [51]. However, renal VEGF expression in the DHFU, DHF, and DHU groups was not up-regulated, and even slightly decreased, which was in agreement with the findings of human diabetic nephropathy. Findings from another study revealed that the expression levels of VEGF mRNA and protein significantly decreased, which showed that a lack of VEGF, rather than an excess of VEGF, caused the progression of human diabetic nephropathy [52].

As the link between host homeostasis and the microbiota, SCFAs play vital roles in regulating glucolipid metabolism, immunity, and intestinal development [53]. Acetic acid is a net product of carbohydrate fermentation in most anaerobic bacteria, whereas butyric and propionic acids are produced from carbohydrate or protein fermentation by a distinct subset of bacteria. Findings from Kindt et al. indicated that the intestinal flora promoted liver fatty acid metabolism by providing a high level of acetate to synthesize stearate and palmitate in mice [54]. Moreover, dietary fructose activates the Ack pathway, which is involved in acetic acid generation, triggering the bacterial stress response and further promoting phage production [55]. In our study, the significantly high content of acetic acid in diabetic hamsters with hyperuricemia and dyslipidemia might be attributed to the combination of a high-fat/cholesterol and high- fructose water diet. As a main energy source for colon epithelial cells, butyric acid plays a crucial role in maintaining the health and integrity of the intestinal mucosa [56]. Butyrate and propionate prevent obesity induced by diet and regulate intestinal hormones [57]. Li et al. reported that the contents of butyric and propanoic acids in fecal samples from hyperuricemic mice were significantly lower than those in samples from normal mice [56]. In agreement with Li’s results, decreased butyric, propanoic and isobutyric acid levels in diabetic hamsters with hyperuricemia indicated that high uric acid could affect the gut microbiota and epithelial integrity in diabetic hamsters.

Previous studies have shown that renal dysfunction in patients with diabetes is closely related to dysbiosis of the intestinal flora [58]. Dysbiosis is generally attributed to a decreased proportion of multiple probiotics and increased pathogenic bacteria. Liang et al. reported that a high-fat/cholesterol diet may affect the gut microbiota, resulting in a sharp decrease in microbial richness and diversity in mice [40]. Inconsistent with Liang’s findings, we found that high-fat/cholesterol diets decreased the richness of the intestinal flora in healthy hamsters but increased the richness of the intestinal flora in diabetic hamsters according to the Chao index. Moreover, the Chao and Shannon indices revealed that the intestinal flora richness and diversity of bacterial communities in diabetic hamsters with hyperuricemia were unrelated to elevated uric acid. However, Ness et al. reported that high gut microbial diversity was related to low uric acid in patients with hyperuricemia [59]. In this study, NMDS and PCoA revealed that the gut microbiota structure in diabetic hamsters with hyperuricemia and dyslipidemia was altered, which may account for the synergistic effects of uric acid and a high-fat/cholesterol diet on the gut microbiota structure in the setting of diabetes. *Firmicutes* and *Bacteroidetes* constitute most microbiota in the human gastrointestinal tract at the phylum level [60]. Moreover, the F/B ratio is a critical indicator of intestinal homeostasis, and a decreased F/B ratio alleviates endotoxemia, inflammation and intestinal permeability [60]. Our findings indicated that the relative abundance of *Firmicutes* decreased and the abundance of *Bacteroidetes* increased in diabetic hamsters, accompanied by a significant decrease in the F/B value, in agreement with Wang’s results that the F/B value in patients with diabetic nephropathy was significantly lower than that in the healthy population. In hyperuricemic hamsters, some dominant bacteria at the genus level (*norank_o_Clostridia_UCG-014*, *norank_f_Muribaculaceae*, *unclassified_f_Lachnospiraceae* and *noran_f_Eubacteriaceae*) were not significantly altered compared with those in normal diabetic hamsters. In a mice model with Alzheimer’s disease, *Allobaculum* is related to improved metabolic health and prolonged lifespan in response to caloric restriction in mice [61]. In our study, the enrichment of *Allobaculum* in hamsters fed a HFCD was significantly reduced after PO treatment, indicating that high uric acid decreased the metabolic health of diabetic hamsters. Wang et al. reported an increased relative abundance of *Lleibacterium* in mice fed high-fat diet and speculated that *Lleibacterium* might be relate to the cause of hyperlipidemia [62]. Our findings revealed that the relative abundance of *Lleibacterium* increased in diabetic hamsters with hyperuricemia and dyslipidemia, indicating that *Lleibacterium* might be involved in enzymes that participated in lipid and purine metabolism.

The gut–kidney axis and intestinal permeability change, leading to dysregulation of the intestinal flora [63]. Moreover, changes in the intestinal flora may also be associated with diet, drug use, and lifestyle in patients with diabetes. The intricate relationship between uric acid metabolism and the gut microbiota involve a bidirectional interaction that can influence both the host’s gut environment and uric acid levels [8]. Our results indicated that the characteristic microbiota in the DHFU group obviously changed, suggesting that alterations in the gut environment affected gut barrier function. We also detected more *Lleibacterium* in diabetic hamsters with hyperuricemia and dyslipidemia, indicating that *Lleibacterium* was positively correlated with lipid metabolic disorders and renal function indicators. However, the role that *Lleibacterium* plays in diabetic renal injury remains unclear. Yuan et al. reported that the serum uric acid level in children with hyperuricemia was positively related to the genera *Streptococcus*, *Morganella*, and *Actinomyces* and negatively related to the genera *Parabacteroides*, *Oscillospira*, *Faecalibacterium*, *Bilophila*, *Alistipes* and *Phascolarctobacterium* [64]. In our study, uric acid levels in diabetic hamsters with hyperuricemia and dyslipidemia were positively associated with *Bacteroides*, *unclassified _f_Ruminococcaceae*, *Candidatus_Saccharimonas*, *norank_f_Muribaculaceae*, *Prevotellaceae_NK3B31_group*, and *Prevotelaceae_UCG-001* and negatively associated with *Helicobacter*, which might be attributed to species differences, STZ induction, and dietary high-fat/cholesterol. Moreover, *unclassified_f_Ruminococcaceae* and *Prevotellaceae_UCG-001* have also been shown to be related to the production of SCFA in db/db mice and hyperglycemic mice, respectively [65,66]. More studies are needed to explain how high uric acid levels affect animal host health through each of these gut flora-associated signaling pathways.

However, several limitations of this need to be pointed out. Hamsters should be housed in metabolic cages for accurately measuring urine, intake of feed and water. The relatively short animal modeling duration should be extended to enable a better observation of the diabetes progression. Potential strain-specific effects in hamsters and comprehensive histopathological assessment also should be considered.

## 5. Conclusions

The current study indicated that serum uric acid levels were jointly elevated by PO treatment and dietary high-fat/cholesterol in the setting of diabetes. Decreased antioxidant capacity in diabetic hamsters with hyperuricemia and dyslipidemia generated some oxidative damage and further stimulated renal inflammation and damage. High uric acid and dietary high-fat/cholesterol synergistically up-regulated the renal gene expression of PAI-1 and TGF-β to accelerate renal injury in diabetic hamsters. Diabetic hamsters with hyperuricemia and dyslipidemia presented histological lesions in glomeruli and renal tubules. High uric acid could affect the gut microbiota and epithelial integrity in diabetes by decreasing butyric, propanoic and isobutyric acid levels. The intestinal flora richness and diversity of bacterial communities in diabetic hamsters with hyperuricemia were weakly correlated with high uric acid but positively correlated with a combination of high uric acid and high TC/TG. Significantly decreased F/B value in diabetic hamsters with hyperuricemia and dyslipidemia was similar to that in patients with diabetic nephropathy. High uric acid reduced the enrichment of *Allobaculum* bacteria, which might deteriorate the health of diabetic hamsters, whereas high uric acid and dietary high-fat/cholesterol significantly increased the relative abundance of *Lleibacterium.* Serum uric acid levels in diabetic hamsters with hyperuricemia and dyslipidemia were positively associated with *Bacteroides*, *unclassified _f_Ruminococcaceae*, *Candidatus_Saccharimonas*, *norank_f_Muribaculaceae*, *Prevotellaceae_NK3B31_group*, and *Prevotelaceae_UCG-001* and negatively associated with *Helicobacter*. Summarily, our findings confirmed that high uric acid affected glucolipid metabolism, accelerated renal damage and disrupted the balance of intestinal flora in diabetic animals with hyperuricemia, which provided a scientific basis for metabolic syndrome prevention and control in diabetes.

## Figures and Tables

**Table 1:** Primer sequences of targeted genes for qPCR.

Genes	Forward	Reverse
TNF-α	AACCTCCTGTCCGCCATCAA	GCACTGAGTCGGTCACCTTTC
SREBP-1c	GCTGCTGACAACGGTAAAAACA	CCAGTCTATGGATGGGCAGTTT
PAI-1	CCGTGGAACCAGAACGAGATT	TGATCATACCTCTGGTGTGCCTC
IL-6	CAAGTCGGAGGTTTGGTTACATATG	CATTGTCCATACAGCCAGGGTT
TGF-β	TGACAGCAAAGACAACGCACTC	TGGAGCTGAAGCAGTAGTTGGTG
VEGF	CCTGGCTTTACTGCTGTACCTCC	CAATAGCTGCGCCGGTAGAC
β-actin	CTTTCTTCGCCGCTCCACA	TGACAATGCCGTGTTCAATGG

Note: TNF-α (tumor necrosis factor-α), SREBP-1c (sterol regulatory element binding protein-1c), PAI-1 (plasminogen activator inhibitor-1), IL-6 (interleukin-6), TGF-β (transforming growth factor-β), and VEGF (vascular endothelial growth factor).

**Table 2:** Serum levels of glucose, insulin, uric acid, TG and TC in the different groups (*n* = 6).

Group	Glu (mmol/L)	Insulin (ng/mL)	UA (μmol/L)	TG (mmol/L)	TC (mmol/L)
C	7.14 ± 1.10	1.66 ± 0.16	199.50 ± 22.76	1.93 ± 0.27	3.50 ± 0.66
CHF	7.98 ± 1.13	1.63 ± 0.12	226.33 ± 30.49	9.70 ± 1.99	20.82 ± 3.61 **
DC	15.68 ± 1.78 **^##^	1.08 ± 0.19 **^##^	240.67 ± 22.85	2.48 ± 0.46	4.83 ± 1.05 ^##^
DHF	15.62 ± 2.74 **^##^	1.12 ± 0.15 **^##^	319.33 ± 48.76 **^##&&^	109.67 ± 15.33 **^##&&^	62.13 ± 15.33 **^##&&^
DHU	17.60 ± 2.44 **^##^	1.13 ± 0.08 **^##^	446.50 ± 27.58 **^##&&$$^	5.53 ± 1.42 ^$$^	4.42 ± 0.89 ^##$$^
DHFU	16.88 ± 2.81 **^##^	1.07 ± 0.12 **^##^	499.50 ± 61.96 **^##&&$$@^	119.88 ± 27.14 **^##&&@@^	72.92 ± 16.62 **^##&&@@^

Note: ** *p* < 0.01 indicates significant differences between C vs. CHF, DC, DHF, DHU, DHFU; ^##^ *p* < 0.01 indicates significant differences between CHF vs. DC, DHF, DHU, DHFU; ^&&^ *p* < 0.01 indicates significant differences between DC vs. DHF, DHU, DHFU; ^$$^ *p* < 0.01 indicates significant differences between DHF vs. DHU, DHFU; ^@^ *p* < 0.05 or ^@@^ *p* < 0.01 indicates significant differences between DHU vs. DHFU.

**Table 3:** BW, Kidney index and liver index of the different groups tested after drug administration (*n* = 6).

Group	BW(g)	Kidney Index(%)	Liver Index(%)
C	153.17 ± 5.08	0.64 ± 0.04	2.90 ± 0.16
CHF	164.00 ± 14.93	0.67 ± 0.05	4.58 ± 0.17 **
DC	146.00 ± 10.32 ^##^	1.19 ± 0.3 **^##^	3.94 ± 0.51 **^#^
DHF	144.67 ± 5.54 ^##^	1.31 ± 0.16 **^##^	7.24 ± 0.82 **^##&&^
DHU	139.00 ± 9.01 *^##^	1.23 ± 0.18 **^##^	3.97 ± 0.25 **^#$$^
DHFU	134.67 ± 14.95 *^##^	1.38 ± 0.22 **^##^	6.12 ± 0.52 **^##&&$$@@^

Note: * *p* < 0.05 or ** *p* < 0.01 indicates significant differences between C vs. CHF, DC, DHF, DHU, DHFU; ^#^ *p* < 0.05 or ^##^ *p* < 0.01 indicates significant differences between CHF vs. DC, DHF, DHU, DHFU; ^&&^ *p* < 0.01 indicates significant differences between DC vs. DHF, DHU, DHFU; ^$$^ *p* < 0.01 indicates significant differences between DHF vs. DHU, DHFU; ^@@^ *p* < 0.01 indicates significant differences between DHU vs. DHFU.

**Table 4:** CRE, BUN, ALT, AST, and ALP levels in the different groups after drug administration (*n* = 6).

Group	CRE (μmol/L)	BUN (mg/dL)	ALT (u/L)	AST (u/L)	ALP (u/L)
C	29.47 ± 2.80	19.28 ± 3.33	107.16 ± 15.47	72.61 ± 11.46	132.03 ± 16.18
CHF	190.38 ± 21.16 **	26.83 ± 3.88 *	217.23 ± 48.17 **	117.97 ± 16.03 **	124.20 ± 15.85
DC	28.59 ± 7.44 ^##^	26.64 ± 4.09 *	109.37 ± 21.65 ^##^	59.43 ± 7.01 ^##^	141.35 ± 17.52
DHF	665.15 ± 71.02 **^##&&^	27.42 ± 7.00 *	126.61 ± 20.08 ^##^	257.99 ± 46.29 **^##&&^	173.06 ± 20.42 **^##&^
DHU	30.03 ± 5.09 ^##$$^	25.98 ± 4.55 *	89.97 ± 7.78 ^##&^	68.21 ± 7.01 ^##$$^	179.86 ± 17.55 **^##&&^
DHFU	920.05 ± 106.26 **^##&&$$@@^	32.82 ± 6.32 **^@^	200.10 ± 27.55 **^&&$$@@^	414.39 ± 23.47 **^##&&$$@@^	217.57 ± 34.34 **^##&&$$@@^

Note: * *p* < 0.05 or ** *p* < 0.01 indicates significant differences between C vs. CHF, DC, DHF, DHU, DHFU; ^##^ *p* < 0.01 indicates significant differences between CHF vs. DC, DHF, DHU, DHFU; ^&^ *p* < 0.05 or ^&&^ *p* < 0.01 indicates significant differences between DC vs. DHF, DHU, DHFU; ^$$^ *p* < 0.01 indicates significant differences between DHF vs. DHU, DHFU; ^@^ *p* < 0.05 or ^@@^ *p* < 0.01 indicates significant differences between DHU vs. DHFU.

**Table 5:** XOD, T-SOD, MDA and GSH-PX of the different groups tested after drug administration (*n* = 6).

Group	XOD (U/gprot)	T-SOD (U/mgprot)	MDA (nmol/mgprot)	GSH-Px (nmol GSH/min/mg Protein)
C	13.31 ± 1.23	281.08 ± 18.33	0.60 ± 0.05	256.99 ± 25.85
CHF	20.45 ± 2.98 **	282.55 ± 18.41	0.79 ± 0.03	229.31 ± 7.84
DC	12.79 ± 1.11 ^##^	309.15 ± 23.42	2.12 ± 0.32 **^##^	245.74 ± 28.99
DHF	23.48 ± 2.0 **^#&&^	263.23 ± 26.09 ^&&^	1.67 ± 0.13 **^##&&^	255.51 ± 18.93
DHU	11.92 ± 1.18 ^##$$^	270.60 ± 29.34 ^&^	1.79 ± 0.20 **^##&&^	250.32 ± 24.37
DHFU	25.73 ± 2.57 ^**##&&@@^	241.88 ± 22.63 *^##&&^	2.35 ± 0.18 **^##&$$@@^	224.32 ± 20.10 *^$^

Note: * *p* < 0.05 or ** *p* < 0.01 indicates significant differences between C vs. CHF, DC, DHF, DHU, DHFU; ^#^ *p* < 0.05 or ^##^ *p* < 0.01 indicates significant differences between CHF vs. DC, DHF, DHU, DHFU; ^&^ *p* < 0.05 or ^&&^ *p* < 0.01 indicates significant differences between DC vs. DHF, DHU, DHFU; ^$^ *p* < 0.05 or ^$$^ *p* < 0.01 indicates significant differences between DHF vs. DHU, DHFU; ^@@^ *p* < 0.01 indicates significant differences between DHU vs. DHFU.

## Data Availability

The raw data is available by the corresponding author upon request.

## Abbreviations

PO: potassium oxonate; IR: insulin resistance; STZ: streptozotocin; CMC-Na: sodium carboxymethyl cellulose; HFCD: high-fat/cholesterol diet; Glu: fasting blood glucose; BW: Body Weight; TC: total cholesterol; TG: triglyceride; CRE: creatinine; BUN: urea nitrogen; ALP: alkaline phosphatase; AST: aspartate transaminase; ALT: alanine transaminase; XOD: Xanthine Oxidase; T-SOD: total superoxide dismutase; GSH-Px: glutathione peroxidase; MDA: malondialdehyde; H&E: hematoxylin and eosin; PAS: Periodic Acid-Schiff stain; TNF-α: tumor necrosis factor-α; SREBP-1c: sterol regulatory element binding protein-1c; PAI-1: plasminogen activator inhibitor-1; IL-6: interleukin-6; TGF-β: transforming growth factor-β; VEGF: vascular endothelial growth factor; SCFA: short-chain fatty acid; ASV: amplicon sequence variant; PCoA: principal coordinate analysis; NMDS: nonmetric multidimensional scaling; LEfSe: LDA effect size; UOX: urate oxidase.
